# Pleiotrophin Overexpression Reduces Adolescent Ethanol Consumption and Modulates Ethanol‐Induced Glial Responses and Changes in the Perineuronal Nets in the Mouse Hippocampus

**DOI:** 10.1111/cns.70159

**Published:** 2024-12-09

**Authors:** Milagros Galán‐Llario, María Rodríguez‐Zapata, Teresa Fontán‐Baselga, Héctor Cañeque‐Rufo, Alba García‐Guerra, Beatriz Fernández, Esther Gramage, Gonzalo Herradón

**Affiliations:** ^1^ Departamento de Ciencias Farmacéuticas y de la Salud, Facultad de Farmacia Universidad san Pablo‐CEU, CEU Universities, Urbanización Montepríncipe Boadilla del Monte Spain; ^2^ Instituto Universitario de Estudios de Las Adicciones Universidad san Pablo‐CEU, CEU Universities, Urbanización Montepríncipe Madrid Spain; ^3^ Red de Investigación en Atención Primaria de Adicciones Instituto de Salud Carlos III, MICINN and FEDER Madrid Spain

**Keywords:** adolescence, alcohol, glia, perineuronal nets, Pleiotrophin

## Abstract

**Aims:**

To investigate whether pleiotrophin (PTN) overexpression influences ethanol consumption during adolescence and its effects on glial responses, neurogenesis, and perineuronal nets (PNNs) in the mouse hippocampus.

**Methods:**

Male and female adolescent transgenic mice with elevated PTN levels (*Ptn*‐Tg) and controls underwent an intermittent access to ethanol (IAE) 2‐bottle choice protocol. Ethanol consumption, PTN levels, neurogenesis, and glial responses were measured in the hippocampus. Immunohistochemistry was used to assess changes in new neurons, microglial and astrocyte populations, and PNNs.

**Results:**

*Ptn*‐Tg mice consumed significantly less ethanol compared to controls, irrespective of sex. Chronic alcohol exposure reduced PTN levels in the hippocampus. PTN overexpression decreased the number of new neurons in the dentate gyrus (DG) and prevented ethanol‐induced microglial activation. *Ptn*‐Tg mice had significantly more astrocytes and fewer PNNs, with a higher percentage of parvalbumin (PV) positive cells surrounded by PNNs under basal conditions. However, ethanol drastically reduced the number of PV+ cells in the DG of *Ptn*‐Tg mice, despite the presence of PNNs.

**Conclusion:**

PTN overexpression reduces adolescent ethanol consumption and influences ethanol‐induced effects on hippocampal neurogenesis, glial responses, and PNN remodeling. These findings underscore the importance of PTN in modulating alcohol‐induced neurotoxicity.

## Graphical abstract

Pleiotrophin (PTN) overexpression reduces adolescent ethanol consumption, prevents ethanol‐induced microglial activation, and alters hippocampal neurogenesis and PNNs remodeling, highlighting PTN's role in alcohol‐induced brain alterations.

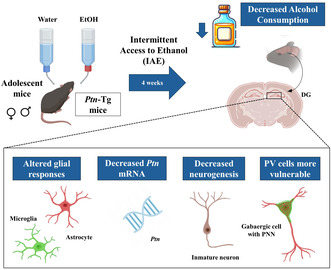

## 1 Introduction

The initiation of alcohol consumption at a young age is of particular concern, with evidence indicating that adolescents as young as 10 or 11 years old are beginning to engage in alcohol consumption [1]. This trend escalates significantly, with a substantial increase in alcohol consumption observed among young adults upon entering college [2]. In addition, adolescents show a reduced sensitivity to the sedative effects of alcohol and tend to underestimate the risks associated with alcohol consumption, which predisposes them to engage in binge drink, a pattern of consumption known to inflict more damage in the developing brain [3]. Moreover, recent statistics highlight a rising trend of binge drink among young women, strengthening the need for researchers to address the adverse effects of alcohol on both sexes [4].

Adolescence represents a pivotal developmental stage, particularly concerning the maturation of the central nervous system. This period is characterized by enhanced myelination processes, alterations in connectivity among brain regions, synaptic pruning, and substantial neurogenesis (see review by [5]). While adult neurogenesis is confined to specific brain regions, namely the subventricular zone and the dentate gyrus (DG), it plays a crucial role in cognitive processing and behaviors requiring plasticity, such as contextual learning and threat identification [6]. Notably, alcohol and other substances have been shown to adversely impact immature neurons following adolescent consumption, with effects that may persist into adulthood [7].

The mechanisms underlying alcohol‐induced neurogenesis impairment are diverse. Recent studies have shed light on the role of the immune response in mediating the consequences of alcohol consumption, stressing the significance of neuroimmune interactions in alcohol‐induced neurotoxicity [8]. Those interactions involve the response of glial cells, including astrocytes and microglia, which trigger neuroinflammation in response to alcohol exposure [9, 10].

Emerging research has also shown the effects of alcohol and other substances on perineuronal nets (PNNs) and how they contribute to the actions of these substances in the brain [11, 12]. PNNs are extracellular matrix structures enveloping neurons, particularly fast‐spiking cells like parvalbumin‐positive neurons. These nets play a critical role in regulating dendritic plasticity, neuronal synchronicity, and protecting neurons from oxidative stress [13].

Collectively, these findings emphasize the need to comprehensively examine the diverse mechanisms involved in alcohol consumption and its neurotoxic effects, with a specific focus on the adolescent brain. In this context, pleiotrophin (PTN) is a cytokine found upregulated in the brain after alcohol administration [14]. PTN is abundantly expressed in many organs during fetal development, while its expression is restricted to specific organs, including the brain, during adulthood [15]. Using transgenic mice with *Ptn* overexpression in the brain, previous studies have shown the implication of PTN in neuroinflammation, potentiating LPS‐induced microglial responses [16], and the influence of this cytokine on cerebral and behavioral responses to amphetamine [17] and behavioral responses to alcohol [14]. PTN has different receptors, being receptor protein tyrosine phosphatase β/ζ (RPTPβ/ζ) of special relevance in the CNS. PTN inhibits the phosphatase activity of RPTPβ/ζ, which is expressed in important areas involved in alcohol effects such as prefrontal cortex (PFC), amygdala, or hippocampus [18, 19]. Furthermore, recent investigations utilizing MY10, an inhibitor of RPTPβ/ζ, have highlighted its role in modulating neuroinflammatory responses and ethanol consumption [20, 21]. Importantly, inhibition of RPTPβ/ζ has been shown to protect neurogenesis in adolescent brains following acute and chronic ethanol exposure [10, 20]. Our most recent findings stress the involvement of RPTPβ/ζ in the structure of PNNs, particularly those in the hippocampus, and their alterations in response to ethanol [22].

Given the actions attributed to both PTN in behavioral responses to ethanol and RPTPβ/ζ in ethanol consumption and its harmful effects on the adolescent brain, investigating the effects of PTN on a model of alcohol intake resembling adolescent drink patterns is of particular interest. In this study, we aim to elucidate PTN‐mediated effects in response to chronic ethanol consumption, examining potential mechanisms beyond RPTPβ/ζ inhibition. Given the diversity of receptors for PTN and of modulatory actions of this cytokine, these findings may identify novel targets for therapeutic interventions aimed at mitigating alcohol‐induced neurotoxicity in the adolescent brain.

## 2 Materials and Methods

### 2.1 Animal Protocol

Transgenic mice overexpressing *Ptn* generated on the C57BL/6J background (*Ptn*‐Tg) were generated as previously described [14]. Male and female C57BL/6J wild type (*Ptn‐*Wt) and *Ptn*‐Tg mice of 5–6 weeks of age (15–22 g) were used. Mice were maintained under controlled environmental conditions (22 ± 1°C and a 12‐h light/12‐h dark cycle) with ad libitum access to food and water. All animal procedures were conducted in accordance with the guidelines outlined in the National Institutes of Health Guide for the Care and Use of Laboratory Animals and the European Union Laboratory Animal Care Rules (2010/63/EU directive). The protocols were approved by the Animal Research Committee of USP‐CEU and by the Comunidad de Madrid (authorization reference: PROEX 76.0/20).

**FIGURE 1:**
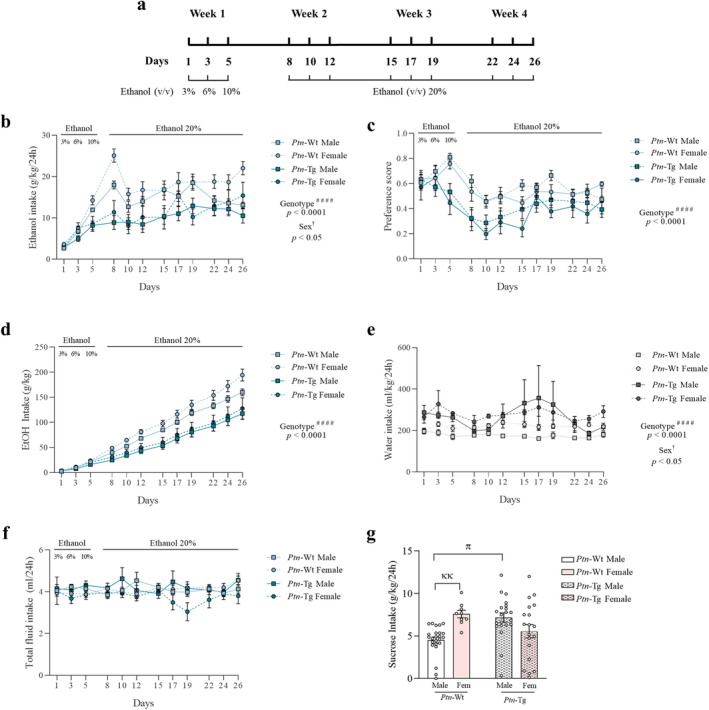
*Ptn* overexpression reduces ethanol consumption during adolescence in an IAE paradigm. Schematic representation of the protocol followed (a). Graphs represent data (mean ± S.E.M.) from ethanol intake after 24 h (b), preference score (c), cumulative ethanol intake (d). water intake (e), total fluid consumption (f), and sucrose intake (g) are also depicted. ^####^ *p* < 0.0001 for significant effect of genotype from three‐way ANOVA. ^†^ *p* < 0.05 for significant effect of sex from three‐way ANOVA. ^κκ^ *p* < 0.01 vs. Female sucrose *Ptn‐*Wt. ^π^ *p* < 0.05 vs. Male sucrose *Ptn‐*Wt.

All animals followed an intermittent access to ethanol (IAE) protocol as previously described [20]. Briefly, during the initial week (Figure 1a), mice were offered a choice between two bottles, one containing ethanol and the other water, with ethanol concentration progressively escalating (3%, 6%, 10% v/v on consecutive drink days) [20]. Subsequently, mice had 24‐h access to a 20% v/v ethanol solution on Monday, Wednesday, and Friday each week for 3 weeks (Figure 1a). As controls, another group of animals followed the same protocol but only being presented with water bottles. Furthermore, to control for general palatable substance intake, a different group of mice was used. They had to choose between a 2% sucrose solution and water on Monday, Wednesday, and Friday during 1 week.

### 2.2 Immunohistochemistry

#### 2.2.1 Tissue Collection

Immediately following the final drink session, mice were anesthetized with pentobarbital and euthanized by transcardial perfusion with 0.9% saline solution, followed by 4% paraformaldehyde. Brains were post‐fixed overnight and transferred to a 30% sucrose solution containing 0.1% sodium azide for storage at 4°C until analysis. Thirty μm sections of the hippocampus were obtained using a sliding microtome (Leica SM2010 R). Immunohistochemical studies were performed on one slice per 360 μm for the dentate gyrus (DG) (from the bregma 2.12 to 3.8 mm).

#### 2.2.2 Immunohistochemical Analysis

All immunohistochemical analyses were conducted as previously described [10]. Briefly, to study doublecortin (DCX) + neurons, sections underwent an antigen retrieval procedure, followed by overnight incubation with rabbit anti‐DCX (Cell Signaling Technology, Danvers, MA; 1:1000) antibody at 4°C, and subsequent incubation with biotinylated secondary antisera (Vector, Burlingame, CA) at RT. The avidin‐biotin reaction was performed using a Vectastain Elite ABC peroxidase kit, and immunoreactivity was visualized using 0.06% diaminobenzidine (DAB) (Sima‐Aldrich, St. Louis, MO) and 0.03% H_2_O_2_ (Sigma‐Aldrich, St. Louis, MO). Photomicrographs were captured with a Leica SCN400 Scan Scanner (Leica, Solms, Germany).

In addition, to assess glial responses, DG sections were incubated overnight at 4°C with mouse anti‐glial fibrillary acidic protein (GFAP; Millipore, Madrid, Spain; 1:1000) and rabbit anti‐ionized calcium‐binding adaptor molecule 1 (Iba1, Wako, Osaka, Japan; 1:1000) antibodies, followed by a 30‐min incubation with the corresponding Alexa‐Fluor‐555 and Alexa‐Fluor‐488 secondary antibodies (Invitrogen, Waltham, MA, USA; 1:500). Perineuronal nets (PNNs) were studied by blocking sections in glycoprotein‐free blocking solution and incubating them with biotinylated *Wisteria floribunda* agglutinin (WFA, Vector Laboratories, CA, USA, 1:2000), followed by incubation with Dylight 488‐conjugated streptavidin (Vector Laboratories, CA, USA; 1:200). WFA serves as a marker for PNNs due to its affinity for *N*‐acetylgalactosamine residues, key components of the extracellular matrix. To visualize parvalbumin (PV) + neurons, sections were re‐blocked with 5% donkey serum for 1 h at RT after WFA staining and then incubated with a PV antibody (Synaptic Systems, Göttingen, Germany; 1:2000) for 24 h at 4°C, followed by incubation with Alexa‐Fluor 555‐conjugated mouse IgG for 1 h at RT (Invitrogen; 1:200). Photomicrographs were captured with a digital camera coupled to an optical microscope (DM5500B, Leica, Solms, Germany) using the LAS X Core software (Leica Microsystems, Wetzlar, Germany; offline version).

#### 2.2.3 Image Analysis

Analysis was conducted using ImageJ/Fiji software (NIH, Bethesda, MD, Version 1.50f) on three photographs of the DG from every subject. Three images (20× objective; size, 640 × 480 μm) from each subject were captured to estimate the number and size of Iba1+ and GFAP+ cells. These images underwent processing using a previously described Fiji macro [10] designed to enhance cellular morphology detection and minimize background artifacts. Subsequently, various parameters were assessed using the “Analyze Particle” function as previously detailed [16].

Additionally, for a more precise examination of microglial morphology, at least 30 Iba1+ cells from two images taken at different sections (40× objective; size, 320 × 240 μm) per experimental condition were analyzed to determine total process length and branching using Sholl's analysis. All cells were traced utilizing the NeuronJ plugin for Fiji, and the total process length of Iba1+ cells was measured. Sholl's analysis, conducted with the ShollAnalysis plugin for Fiji, involved the creation of concentric circles with a spacing of 1 μm between them, marking the number of branches crossing each circle. Plotting the number of crossings against the distance to the microglial body provided a representation of microglial process complexity.

Furthermore, for DCX analysis, three images (10× objective; size, 1280 × 960 μm) were employed to obtain a comprehensive view of the DG. Images were processed as previously described using a Fiji Macro [10]. DCX+ staining within the granular cell layer (GCL) was quantified using the “Threshold” option in ImageJ/Fiji software, with thresholds adjusted using an automatic iterative method provided by ImageJ software. DCX+ density was expressed as a percentage of DCX+ staining in the GCL.

Lastly, for PNNs analysis, two images (10× objective; size, 1280 × 960 μm) from each subject within the DG area were utilized. The analysis was conducted using Polygon AI software, employing an adapted version of a previously detailed protocol to study WFA fluorescence intensity [22]. The number of total PV+ cells, and the intensity and density of WFA+ PNNs, and PV+ neurons both surrounded (WFA+/PV+) and devoid (WFA−/PV+) of WFA coating (cells/mm^3^) within the GCL of the DG was measured in each image. These values were then multiplied by the thickness of the slide to obtain the GCL volume (reference volume) of the image. Finally, the total number of positive cells counted was divided by the reference volume.

The values depicted in the graphs represent the analysis conducted for each image.

### 2.3 Quantitative Real‐Time PCR

Total RNA was isolated from hippocampal tissue using the Total RNA Isolation Kit (Nzytech, Lisbon, Portugal), and first‐strand cDNA was synthesized using the first‐strand cDNA Synthesis Kit (Nzytech). Quantitative real‐time PCR analysis was performed using the SYBR green method (Quantimix Easy kit, Biotools, Madrid, Spain) in a CFX96 Real‐Time System (Bio‐Rad, Hercules, CA, USA). The relative expression of *Ptn* in hippocampi from mice of all experimental groups was normalized using *Rpl13* and *Hprt1* as housekeeping genes, and the data were analyzed by the Livak method. The primer sequences are shown in Table 1.

**TABLE 1:** Primer sets used for qPCR analysis.

Gene	Primer forward	Primer reverse
*Hprt1*	5′‐TGCTCGAGATGTCATGAAGG‐3′	5′‐TATGTCCCCCGTTGACTGAT‐3′
*Rpl13*	5′‐GGTGCCCTACAGTTAGATACCAC‐3′	5′‐TTTGTTTCGCCTCCTTGGGTC‐3′
*Ptn*	5′‐TTGGGGAGAATGTGACCTCAATAC‐3′	5′‐GGCTTGGAGATGGTGACAGTTTTC‐3′

Abbreviations: *Hprt1*, Hypoxanthine phosphoribosyltransferase 1; *Rpl13*, Ribosomal Protein L13; *Ptn*, Pleiotrophin.

### 2.4 Statistics

Statistical analyses were performed using GraphPad Prism version 8 (San Diego, CA, USA). Data are presented as mean ± standard error of the mean (SEM). Ethanol intake (g/kg), total fluid intake (mL), ethanol preference (%) from the IAE assays were analyzed using a three‐way repeated measures (RM) ANOVA considering sex, genotype, and drink session as variables. Sucrose intake (g/kg) data were collapsed across the 3 days and analyzed using a two‐way ANOVA considering sex and genotype as variables. Data from immunohistochemistry studies and gene analysis were analyzed using three‐way ANOVA considering sex, genotype, and ethanol drink as variables. When appropriate, a two‐way ANOVA was employed to dissect the effect of individual variables. Differences were analyzed by post hoc comparisons with Bonferroni's post hoc tests. To compare double‐labeled (WFA+/PV+) populations percentages a Chi‐squared test (χ^2^) was performed to evaluate the distribution among experimental groups. All references to statistical significance made to the three‐way ANOVA test's individual factors or their interaction are detailed in Table S1.

## 3 Results

### 3.1 *Ptn* Overexpression Reduces Alcohol Consumption

Both *Ptn*‐Tg mice and *Ptn‐*Wt male and female mice aged 5–6 weeks were subjected to a 4‐week IAE protocol to assess ethanol consumption (Figure 1). Three‐way ANOVA revealed that *Ptn*‐Tg mice drank significantly less alcohol than their wild‐type counterparts (Figure 1b; *F*_1,61_ = 19.45, *p* < 0.0001) with significant sex differences (Figure 1b; *F*_1,61_ = 4.071, *p* = 0.0480). The two‐way ANOVA within each genotype revealed that *Ptn‐*Wt female mice drank more than males (Figure 1b; *F*_1,34_ = 8.158, *p* = 0.0073), whereas no sex difference (Figure 1b; *F*_1,49_ = 0.8336, *p* = 0.3657) was found in *Ptn*‐Tg mice. Furthermore, the preference score, a measure of alcohol bottle preference over water, was lower in *Ptn*‐Tg mice (Figure 1c; *F*_1,61_ = 16.82, *p* = 0.0001). We also analyzed the total cumulative ethanol consumption over the 4‐week period. The three‐way ANOVA confirmed that *Ptn‐*Wt mice consumed more ethanol at the end of the experiment than *Ptn‐*Tg mice (Figure 1d; *F*_1,61_ = 5.705, *p* < 0.0001). In control experiments, we observed that *Ptn*‐Tg mice consumed significantly more water than *Ptn‐*Wt mice when they had access to two bottles of water for 4 weeks (Figure 1e; *F*_1,49_ = 25.43, *p* < 0.0001). However, no genotypic difference was found when we measured the total fluid consumption of these animals adding up water and ethanol drink (Figure 1f; *F*_1,61_ = 0.5729, *p* = 0.4520). Moreover, to assess whether the preference for a palatable substance is affected by *Ptn* overexpression, we analyzed 2% sucrose intake across a cumulative 3‐day period. Two‐way ANOVA revealed a significant interaction between sex and genotype (Figure 1g; *F*_1,69_ = 14.12; *p* = 0.0004). Post hoc comparisons showed that *Ptn‐*Wt females consumed more sucrose than *Ptn‐*Wt males (*p* = 0.0139), while *Ptn*‐Tg males exhibited higher sucrose intake than their wild‐type counterparts (*p* = 0.0036). Overall, these data indicate that *Ptn* overexpression reduces ethanol consumption in a sex‐independent manner.

### 3.2 Ethanol Consumption Decreases *Ptn* mRNA Levels in the Hippocampus

**FIGURE 2:**
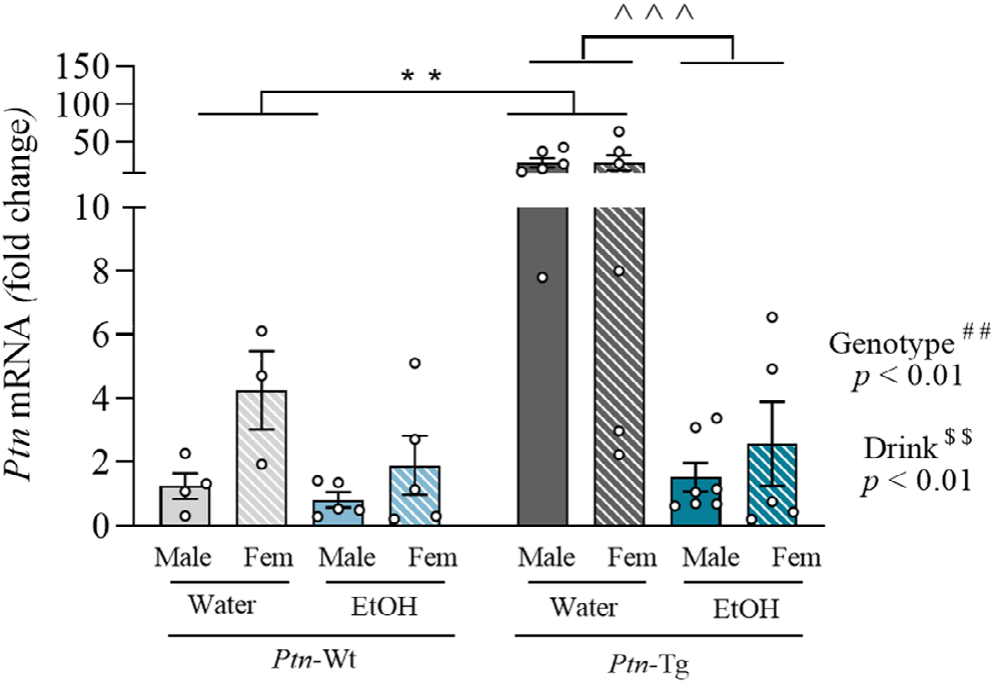
Ethanol consumption decreases *Ptn* levels in the hippocampus. Graph represents data (mean ± S.E.M.) from the quantification of *Ptn* mRNA levels in the hippocampus of *Ptn‐*Wt and *Ptn*‐Tg mice after the IAE protocol. ^##^ *p* < 0.01 for significant effect of genotype from the three‐way ANOVA. ^$$^ *p* < 0.01 for significant effect of drink. ^∧∧∧^ *p <* 0.001 for significant effect of drink excluding the sex variable. **p < 0.01 for significant effect of genotype excluding the sex variable.

The expression levels of *Ptn* were examined in the hippocampus of mice that underwent the IAE procedure during adolescence (Figure 2). As expected, *Ptn* levels were significantly higher in *Ptn*‐Tg brains (*F*_1,34_ = 9.338, *p* = 0.0042). Surprisingly, we found that ethanol decreased significantly the levels of *Ptn* mRNA in the hippocampus, as the three‐way ANOVA revealed a significant effect of the drink variable (*F*_1,34_ = 7.604, *p* = 0.0093). The analysis also revealed a significant interaction between genotype and drink variables (*F*_1,34_ = 5.660, *p* = 0.0231). The subsequent two‐way ANOVA performed excluding the sex variable revealed a significant difference between water groups of both genotypes. Furthermore, it revealed that the reduction of *Ptn* levels induced by ethanol was especially significant in the hippocampus of *Ptn*‐Tg mice (*F*_1,20_ = 4.599, *p* = 0.0445). These results highlight the consequences of ethanol consumption during adolescence on hippocampal *Ptn* levels.

### 3.3 *Ptn* Overexpression and IAE Diminish the Population of Neuronal Progenitors in the Dentate Gyrus

**FIGURE 3:**
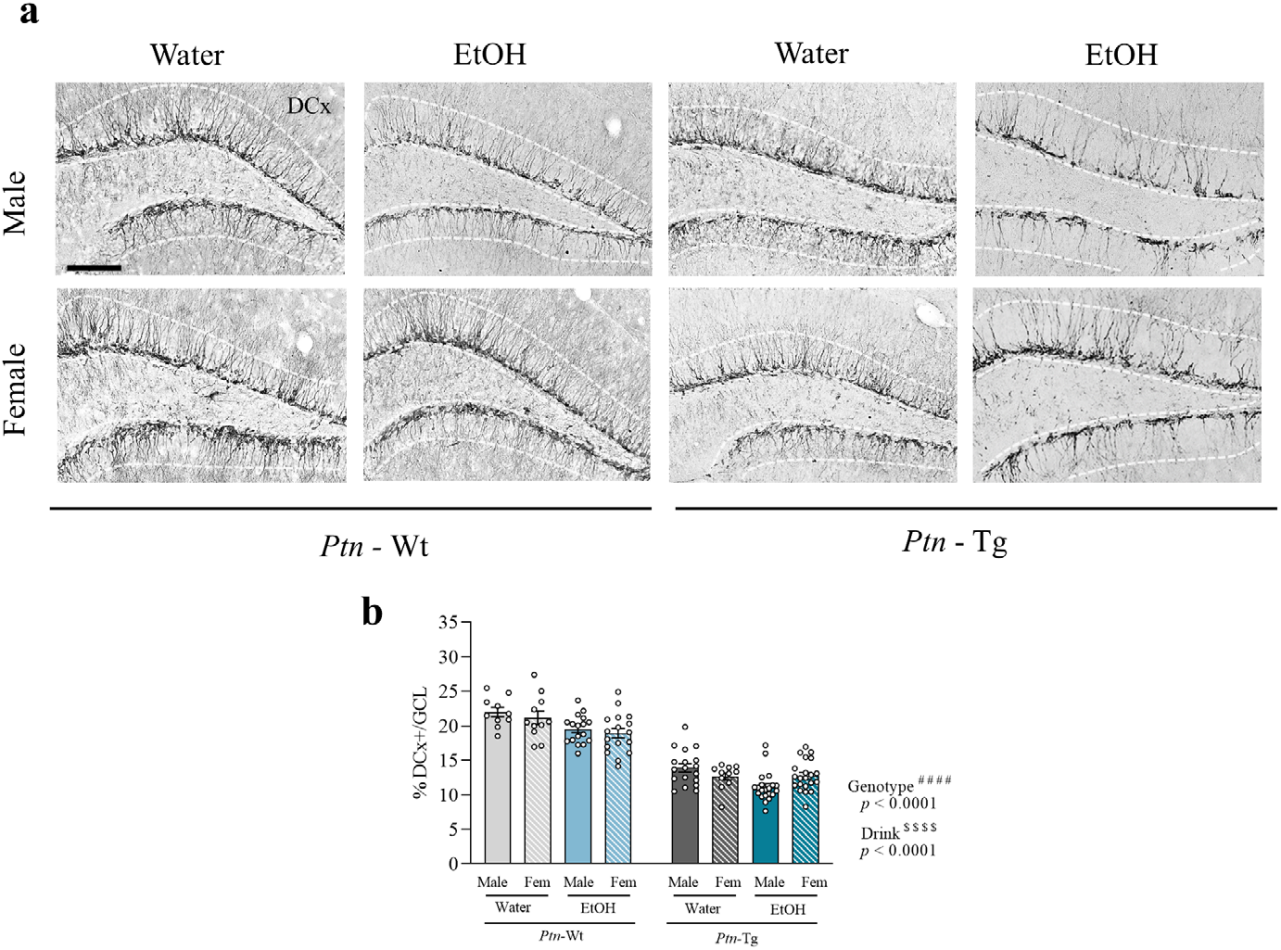
Neurogenesis in the dentate gyrus of mice after adolescent IAE. Photomicrographs are from doublecortin (DCX)‐immunostained hippocampal sections of *Ptn‐*Wt and *Ptn*‐Tg male and female mice. Dashed lines indicate the outline of the granule cell layer of the hippocampal dentate gyrus (a). Graph represents data (mean ± S.E.M.) from the quantification of %DCX positive staining in the granular cell layer (b). ^####^ *p* < 0.0001 for significant effect of genotype from the three‐way ANOVA. ^$$$$^ *p* < 0.0001 for significant effect of drink from the three‐way ANOVA. Scale bar = 200 μm.

The three‐way ANOVA of the DCX+ staining (Figure 3a) revealed a significant effect of ethanol drink (*F*_1,118_ = 17.30, *p* < 0.0001) and of the genotype (*F*_1,118_ = 303.8, *p* < 0.0001) on the percentage of neural progenitors in the GCL (Figure 3b), whereas no differences between sexes were detected (*F*_1,118_ = 0.3399, *p* = 0.5610). We observed a significantly lower percentage of neural progenitors in mice that drunk ethanol. Interestingly, both water and ethanol‐drinking *Ptn*‐Tg mice exhibited significantly fewer neuronal progenitors than *Ptn‐*Wt mice (Figure 3b).

### 3.4 *Ptn* Overexpression Prevents Ethanol‐Induced Microglial Morphological Changes While It Increases Astrocyte Vulnerability to Ethanol

**FIGURE 4:**
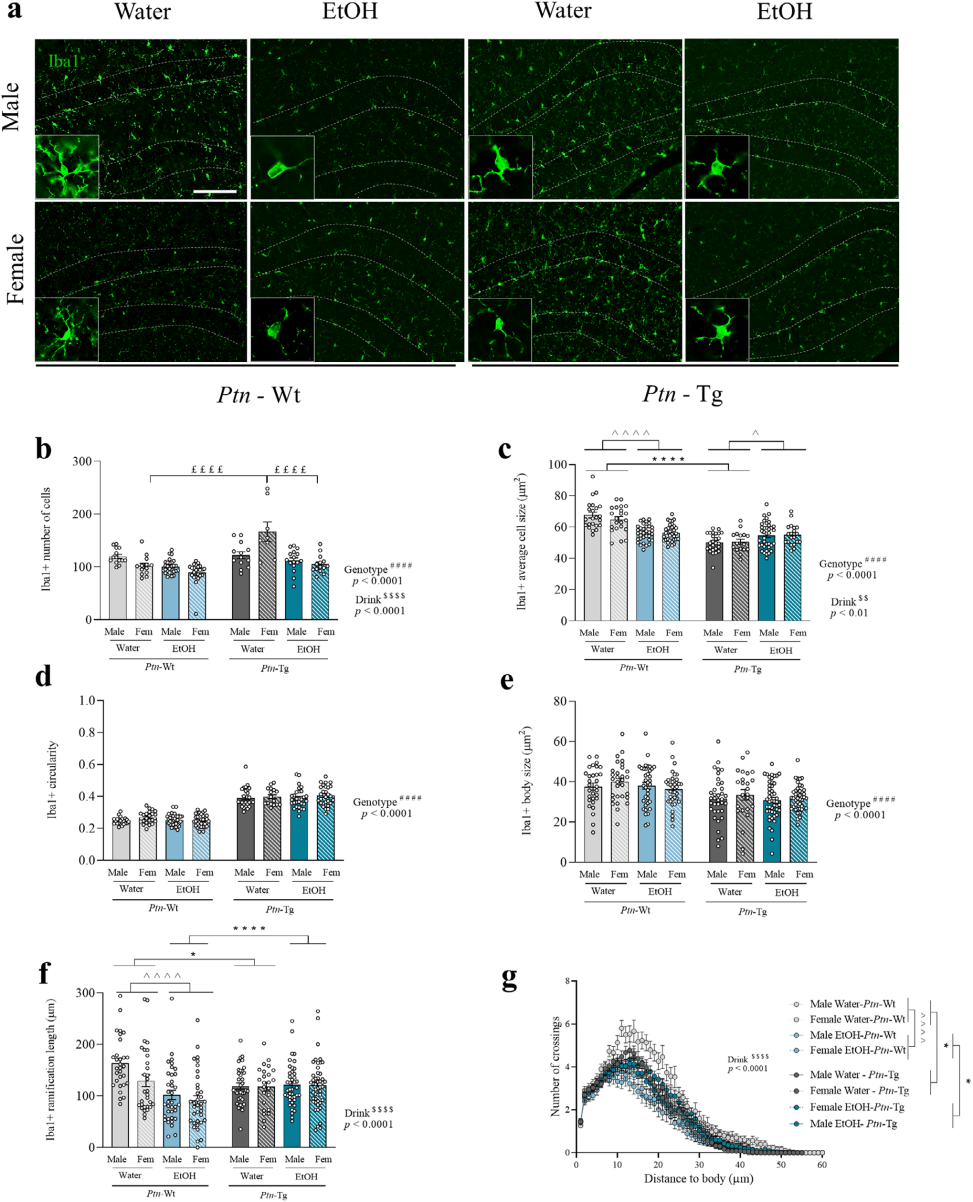
Effects of IAE and *Ptn* overexpression on microglial responses in the adolescent hippocampus. Photomicrographs are from Iba1‐immunostained hippocampal sections of *Ptn‐*Wt and *Ptn*‐Tg male and female mice. Dashed lines indicate the outline of the granule cell layer of the hippocampal dentate gyrus. In the lower left corner, we present a magnified image of a single microglia cell (a). Graphs represent data (mean ± S.E.M.) from the quantification of Iba1 + number of cells (b) in the granular cell layer. Iba 1 + average cell size (c) and circularity (d) are also shown. Graphs depicting Iba1 + body size (e), ramification length (f), and ramification complexity of the Iba1+ cells (g) in this area are also presented. ^####^ *p* < 0.0001 for significant effect of genotype. $$ *p* < 0.01; $$$$ *p* < 0.0001" replacing $$$ *p* < 0.001 for significant effect of drink. ^^^ *p* < 0.05; ^^^^^^ *p* < 0.0001 for significant effect of drink excluding sex variable. *p < 0.05; ****p < 0.0001 for significant effect of genotype excluding sex variable. ^££££^ *p* < 0.0001 vs. Female Water *Ptn*‐Tg. Scale bar = 100 μm.

We investigated microglial morphological changes in the DG of *Ptn‐*Tg *and Ptn‐*Wt mice subjected to IAE during adolescence (Figure 4). Three‐way ANOVA revealed a significant effect of the genotype (*F*_1,120_ = 33.26, *p* < 0.0001), of ethanol drink (*F*_1,120_ = 40.55, *p* < 0.0001) and a significant interaction of sex, drink, and genotype, on the number of Iba1+ cells (*F*_1,120_ = 12.92, *p* = 0.0005) (Figure 4b). Post hoc analysis revealed that the number of Iba1+ cells in the DG of water drink female *Ptn*‐Tg mice was significantly higher than that in *Ptn‐*Wt mice (Figure 4b), accounting for the more pronounced decrease of Iba1+ cells caused by ethanol consumption in female *Ptn*‐Tg mice. Regarding Iba1+ cell size (Figure 4c), the three‐way ANOVA showed that *Ptn‐*Tg mice had significantly smaller microglial cells than *Ptn‐*Wt mice (*F*_1,206_ = 75.33, *p* < 0.0001). We did not find a significant effect of sex (*F*_1,206_ = 0.2212, *p* = 0.6886). However, a significant effect of ethanol on the size of microglia (*F*_1,206_ = 7.743, *p* = 0.0059) and a significant interaction between genotype and ethanol drink were found (*F*_1,206_ = 51.53, *p* < 0.0001). Thus, we performed a two‐way ANOVA excluding the sex variable, which revealed that microglial cells are smaller in the DG of *Ptn*‐Tg mice in the basal state. In addition, ethanol significantly reduced the size of microglia only in *Ptn‐*Wt mice, while it did the opposite in *Ptn*‐Tg mice (Figure 4c). Circularity (Figure 4d), a marker of activation, showed a significant effect of the genotype (*F*_1,206_ = 430.8, *p* < 0.0001), revealing a microglia circularity index significantly higher in the DG of *Ptn*‐Tg mice independently of sex or ethanol consumption. We also observed a significant effect of the genotype on the Iba1+ body size (*F*_1,274_ = 25.53, *p* < 0.0001), which was smaller in *Ptn*‐Tg mice (Figure 4e). Finally, we studied the ramification length and complexity of microglial cells through Sholl analysis. Three‐way ANOVA showed a significant effect of ethanol drink (*F*_1,274_ = 15.77, *p* < 0.0001) on ramification length (Figure 4f) and a significant interaction between genotype and drink (*F*_1,274_ = 19.37, *p* < 0.0001). The subsequent two‐way ANOVA highlighted that *Ptn*‐Tg microglia branch length was significantly different from their wild‐type counterparts, especially in the case of the mice that underwent IAE. Ethanol consumption decreased the length of branches only in *Ptn‐*Wt mice (Figure 4f). When studying ramification complexity, the three‐way ANOVA only revealed a significant effect of drink (Figure 4g; *F*_1,274_ = 15.708, *p* < 0.0001) and of the interaction between genotype and drink (*F*_1,274_ = 19.292, *p* < 0.0001). The two‐way ANOVA excluding the sex variable showed similar results to those obtained in the analysis of branch length. First, the branching scheme from *Ptn*‐Tg microglial cells was significantly different from the one presented by *Ptn‐*Wt cells, comparing mice with the same drink protocol. Furthermore, microglial cells of water drink *Ptn‐*Wt mice were significantly more complex than in the rest of the groups. Ethanol reduced the complexity of microglia of *Ptn‐*Wt mice but had a lesser effect on *Ptn*‐Tg mice. These results suggest a more pronounced effect of ethanol on microglial cells of *Ptn‐*Wt mice, indicating that *Ptn* overexpression prevents ethanol‐induced alterations in microglia.

**FIGURE 5:**
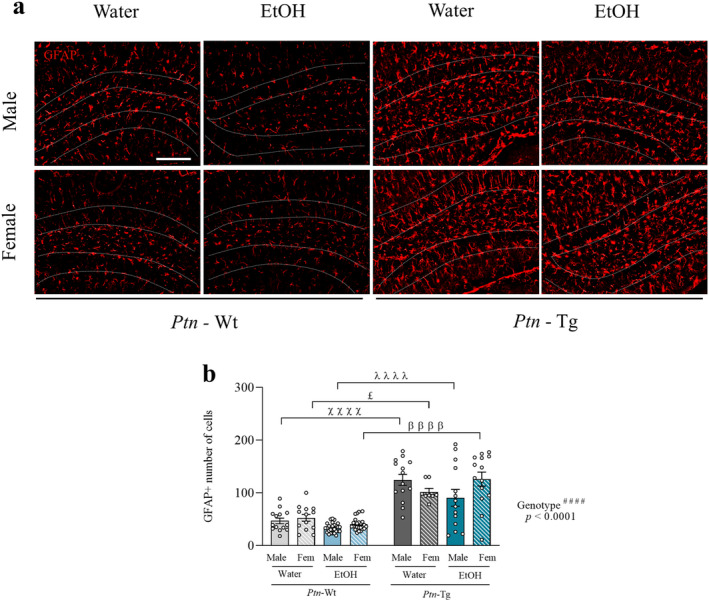
Effects of IAE and *Ptn* overexpression on astrocytic responses in the adolescent hippocampus. Photomicrographs are from GFAP‐immunostained hippocampal sections of Astrocyte cells in dentate gyrus of *Ptn‐*Wt and *Ptn*‐Tg male and female mice. Dashed lines indicate the outline of the granule cell layer of the hippocampal dentate gyrus (a). Graph represents data (mean ± S.E.M.) from the quantification of GFAP + number of cells (b) in the granular cell layer. ^####^ *p* < 0.0001 for significant effect of genotype. ^£^ *p* < 0.05 vs. Female Water *Ptn*‐Tg. ^ᵝᵝᵝᵝ^ *p* < 0.0001 vs. Female EtOH *Ptn‐*Wt. ^ᵡᵡᵡᵡ^ *p* < 0.0001 vs. Male Water *Ptn‐*Wt. ^λλλλ^ *p* < 0.0001 vs. Male EtOH *Ptn‐*Wt. Scale bar = 100 μm.

Concerning the astrocytic changes induced by ethanol consumption (Figure 5), three‐way ANOVA revealed a significant effect of genotype on astrocytes (*F*_1,118_ = 127.8, *p* < 0.0001), indicating that *Ptn*‐Tg mice presented enhanced astrocytosis (Figure 5b). The analysis also showed a significant interaction between sex, drink, and genotype (*F*_1,118_ = 5.578, *p* = 0.0198). The post hoc analysis revealed significant differences between each experimental group of *Ptn*‐Tg mice and their corresponding controls. Thus, these results suggest that *Ptn* overexpression promotes astrocytosis independently of sex and drink.

### 3.5 Overexpression of *Ptn* Modulates PNNs and Makes PV+ Cells More Vulnerable to Ethanol

**FIGURE 6:**
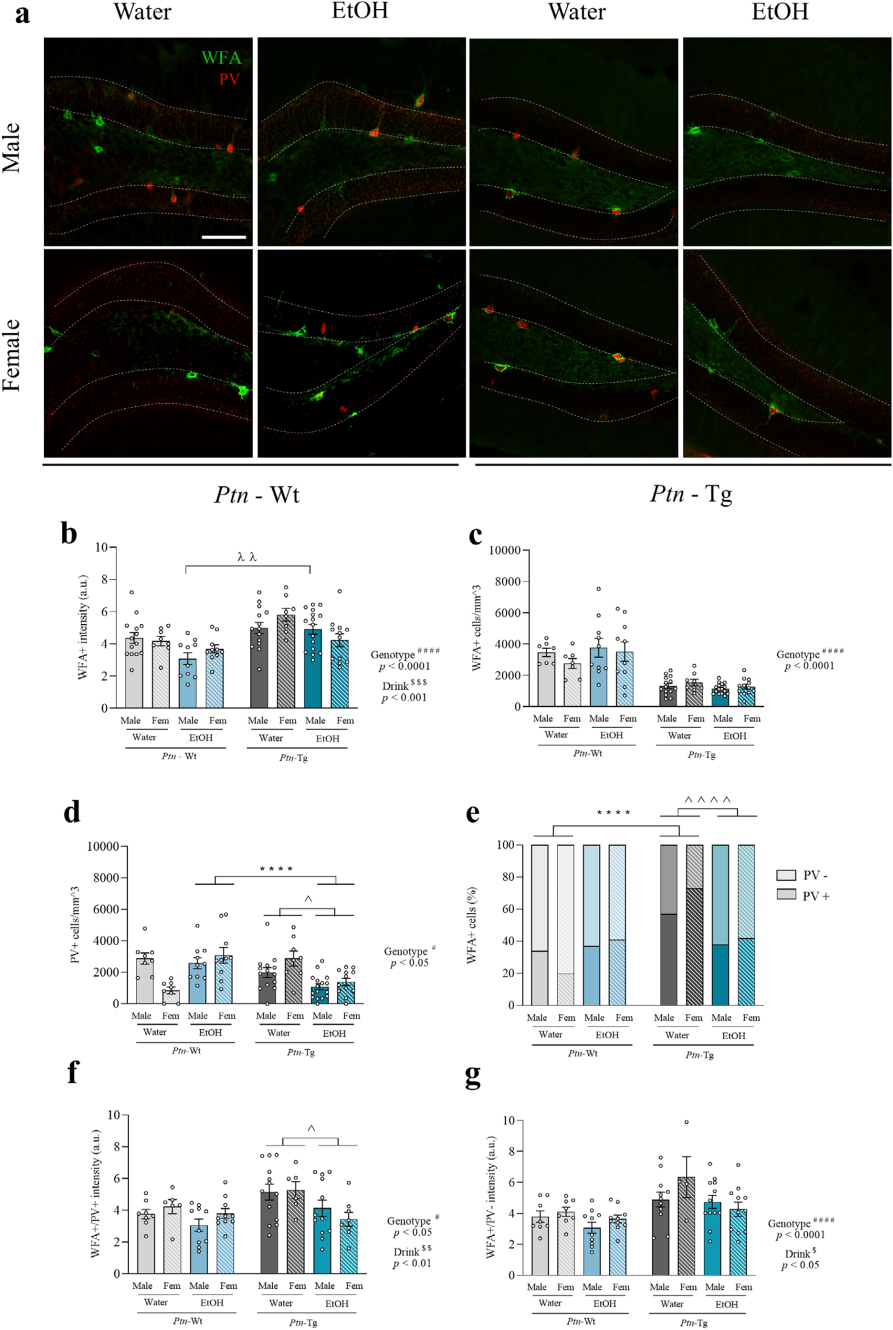
Effects of IAE and *Ptn* overexpression on PNNs and PV+ cells in the adolescent hippocampus. Photomicrographs of WFA (green) and PV fluorescence (red) binding from *Ptn‐*Wt and *Ptn*‐Tg male and female mice. Dashed lines indicate the outline of the granule cell layer of the hippocampal dentate gyrus (a). Graphs represent data (mean ± S.E.M.) from the quantification of WFA intensity (b) and number of WFA+ cells in the granular cell layer (c). Quantification of PV+ cells in this area (d) and graph representing the percentage of WFA+ cells that do or do not colocalize with PV are also shown (e). Graph representing data from the quantification of WFA intensity of PNNs surrounding PV+ neurons (f) and PV− neurons (g). ^#^ *p* < 0.05; ^####^ *p* < 0.0001 for significant effect of genotype. ^$^ *p* < 0.05; ^$$^ *p* < 0.01; ^$$$^ *p* < 0.001 for significant effect of drink. ^^^ *p* < 0.05; ^^^^^^ *p* < 0.0001 for significant effect of drink excluding sex variable. ****p < 0.0001 for significant effect of genotype excluding sex variable. ^λλ^ *p* < 0.01 vs. Male EtOH *Ptn‐*Wt. Scale bar = 100 μm.

As PNNs are structures known to provide neuronal protection and to be influenced by drug consumption, investigating these nets in the context of adolescent IAE drink in *Ptn*‐Tg and *Ptn‐*Wt mice was of interest (Figure 6). Firstly, three‐way ANOVA revealed a significant effect of the genotype on WFA intensity (Figure 6b; *F*_1,84_ = 20.59, *p* < 0.0001), showing more intense PNNs in *Ptn*‐Tg mice. We also observed a significant effect of ethanol drink on PNNs intensity (*F*_1,84_ = 11.27, *p* = 0.0012), showing that ethanol decreases the intensity of PNNs in both genotypes (Figure 6b). In addition, a significant interaction between sex, genotype, and drink was found (*F*_1,84_ = 5.101, *p* = 0.0265). Ethanol‐drinking females from both genotypes tended to show a lower intensity of PNNs than water drink females. In contrast, whereas ethanol‐drinking male *Ptn‐*Wt mice showed decreased intensity of PNNs, ethanol drink *Ptn*‐Tg male mice did not show any alterations, being the intensity of PNNs of ethanol drink *Ptn*‐Tg male mice significantly higher than that in male *Ptn* ^+/+^ mice (Figure 6b).

Three‐way ANOVA of the number of PNNs in the DG revealed a significant effect of genotype (Figure 6c; *F*_1,76_ = 78.19, *p* < 0.0001), showing fewer PNNs in the DG of *Ptn*‐Tg mice than in *Ptn‐*Wt mice in this area. However, we did not detect significant effects of drink (*F*_1,76_ = 0.3881, *p* = 0.5352) or sex (*F*_1,76_ = 0.4098, *p* = 0.5240) on the number of PNNs. As PNNs mostly surround parvalbumin (PV) cells, we studied the number of PV+ cells (Figure 4d). We observed a significant effect of the genotype (*F*_1,78_ = 4.621, *p* = 0.0347), a significant interaction of genotype and drink (*F*_1,78_ = 20.71, *p* < 0.0001), of sex and genotype (*F*_1,78_ = 8.183, *p* = 0.0054) and of the interaction between the three variables (*F*_1,78_ = 10.70, *p* = 0.0016) on the number of PV+ cells. The subsequent two‐way ANOVA within each genotype highlighted that ethanol induced a significant reduction of PV+ cells in *Ptn*‐overexpressing mice (*F*_1,46_ = 16.66, *p* = 0.0002). Accordingly, ethanol drink *Ptn‐*Wt mice showed a significantly higher number of PV cells than ethanol drink *Ptn*‐Tg mice (Figure 6d). Interestingly, when studying the proportion of PNNs that surrounded PV+ neurons, we observed that the proportion of WFA+ cells surrounding PV+ cells was higher in *Ptn*‐overexpressing mice (d.f. (1), χ^2^ = 58.13, *p* < 0.0001) (Figure 6e); However, after IAE, we observed a significant decrease in the proportion of PNNs colocalizing with PV+ cells in the DG of *Ptn*‐Tg mice (d.f. (1), χ^2^ = 25.06, *p* < 0.0001), suggesting that alcohol reduces significantly PV+ cells surrounded by PNNs in *Ptn*‐Tg mice (Figure 6e). To assess whether PNN intensity varied between PV+ and PV− cell populations, we analyzed WFA+ staining intensity separately for each group. A three‐way ANOVA revealed that the intensity of WFA surrounding PV+ cells was significantly influenced by both genotype (Figure 1f; *F*_*1*,65_ = 5.38, *p* = 0.0235) and drink factors (Figure 1f; *F*_*1*,65_ = 8.724, *p* = 0.0044). Similarly, for WFA+/PV− cells, both genotype (Figure 1g; *F*_*1*,66_ = 17.59, *p* < 0.0001) and drink factors (Figure 1g; *F*_*1*,66_ = 6.397, *p* = 0.0138) significantly affected PNN intensity. Alcohol consumption reduced the intensity of PNNs specifically around PV+ neurons in *Ptn*‐Tg mice (Figure 1f; *F*_*1*,35_ = 6.756, *p* = 0.0136), whereas the intensity of PNNs surrounding other cell types (WFA+/PV−) was not significantly altered (Figure 1g; *F*_*1*,34_ = 3.502, *p* = 0.0699).

## 4 Discussion

It has been previously shown that a single ethanol administration upregulates *Ptn* levels in the mouse brain [14]. However, it had not been explored yet the consequences of the increased expression of this cytokine on ethanol consumption and cerebral alterations induced by this drug. Thus, we aimed to study the role of *Ptn* overexpression on ethanol consumption, and its modulatory actions on ethanol effects in the brain including neurotoxicity, glial responses, and PNNs remodeling.

Firstly, we demonstrate for the first time that *Ptn* overexpression reduces ethanol consumption in an IAE paradigm in both male and female adolescent mice. In addition, we observed that *Ptn*‐Tg male mice, not females, exhibited higher sucrose intake than their wild‐type counterparts. Although this control for palatable substance intake was performed over 1 week, compared to the 4 weeks of the IAE procedure, the data support that the effect of *Ptn* overexpression is specific to ethanol because it did not reduce sucrose consumption.

This is remarkable as our previous work using the same protocol showed that MY10, a small molecule inhibitor of the PTN receptor RPTPβ/ζ, also reduced ethanol consumption but only in male adolescent mice [20]. Pleiotrophin has different receptors, one of which is RPTPβ/ζ [23]. PTN acts as an endogenous inhibitor of RPTPβ/ζ, a receptor widely expressed in the brain, including the rewarding circuitry [18]. The data suggest that the mechanism by which *Ptn* modulates alcohol consumption in the male brain involves RPTPβ/ζ receptor blockade, as ethanol consumption reduction occurs both when RPTPβ/ζ is inhibited by *Ptn* overexpression and when it is selectively targeted by exogenous administration of MY10. However, in females, we only observe this drink reduction effect as a result of *Ptn* overexpression, suggesting that the modulation of ethanol consumption by PTN may involve other known receptors of this cytokine in the female brain such as anaplastic lymphoma kinase (ALK), Syndecan‐3, and Neurocan [24].

Interestingly, *Ptn* levels were significantly reduced after chronic ethanol drink in the hippocampi of mice from both genotypes. In contrast, we previously showed that *Ptn* levels are upregulated in the mouse prefrontal cortex (PFC) 1 h after a single administration of a moderate dose of ethanol (2 g/kg) [14]. However, we recently showed that *Ptn* levels are not modulated in the mouse PFC 18 h after a single administration of a very high dose of ethanol (6 g/kg) [21]. Thus, moderate amounts of ethanol increase the expression levels of *Ptn*, while this increase of *Ptn* levels in response to acute and moderate ethanol administrations is not observed after chronic consumption or administrations of higher doses of ethanol, indicating that this homeostatic mechanism against ethanol is lost with chronicity and escalation of this drug's dose.

Given that the adolescent brain is highly plastic, with processes like neurogenesis being exacerbated, we aimed to study neurogenesis after IAE in the context of *Ptn* overexpression. Surprisingly, we found that *Ptn‐*Tg mice have fewer DCX positive neurons than *Ptn‐*Wt mice. Furthermore, although MY10 was shown to prevent ethanol‐induced hippocampal neurogenic loss [10], we do not observe the same protection exerted by *Ptn* overexpression. Recent work by Li et al. [25] concluded that hippocampal PTN infusion rescued the loss of neurogenesis in mice with *Ptn* gene silencing. The apparent contradictory results may be explained by substantial differences between the experimental models, i.e., constitutive *Ptn* overexpression compared to a punctual PTN infusion. All in all, additional studies are needed to clarify the conditions in which added PTN exerts beneficial effects on hippocampal neurogenesis.

Stem cells in the brain are known to produce both astrocytes and glutamatergic neurons, so we studied the astrocytic responses in the hippocampus [26]. While ethanol decreased astrocyte numbers in the DG of both male and female wild‐type mice, it only caused this effect in male *Ptn*‐Tg mice. In contrast, *Ptn‐*Tg females showed increased astrocyte numbers after the IAE protocol, suggesting a sex‐dependent astrocytic response to chronic drink in the presence of high levels of *Ptn*. More interestingly, we found that *Ptn*‐Tg mice showed increased hippocampal astrocytosis compared to *Ptn‐*Wt mice. This effect seems to be region‐specific as previous work from our lab did not render differences in the GFAP+ astrocytes in the PFC or striatum of *Ptn*‐Tg mice. It should be noted that PTN acts on neural stem cells, which may result in both astrogenesis and neurogenesis [25]. Thus, it is tempting to speculate that, in the context of PTN overexpression, the astrocytic lineage from neural stem cells may be favored over the neuronal lineage, as *Ptn*‐Tg mice show basal astrocytosis and fewer neuronal progenitors compared to *Ptn‐*Wt mice.

As alcohol is known to induce a neuroinflammatory response [27], we also studied microglial responses in the dentate gyrus. Remarkably, female *Ptn*‐Tg mice presented increased microgliosis compared to the other experimental groups. More interestingly, in the DG of *Ptn*‐Tg mice, we found smaller microglial cells that were more circular, and with smaller body sizes and fewer branches than microglial cells of *Ptn‐*Wt mice. In addition, we found that *Ptn* overexpression prevents the decrease in branch length and branch complexity observed after IAE in *Ptn‐*Wt mice. This retraction in branches indicates microglial activation after ethanol exposure in *Ptn‐*Wt mice, a response completely ablated by *Ptn* overexpression. Interestingly, other inflammatory insults, such as LPS, produce more evident microglial changes in the PFC of *Ptn*‐Tg mice [16], indicating that the modulation of microglial responses by *Ptn* overexpression depends on the insult.

As PNNs, key extracellular matrix structures for neuronal protection [13] are heavily impacted by drugs of abuse [28], and RPTPβ/ζ plays a significant role in the integrity of PNNs [29], it was of interest to study the potential role of *Ptn* overexpression on ethanol‐induced alterations on PNNs. Using this IAE paradigm, we previously showed that ethanol consumption decreases the intensity of PNNs [22]. In the case of *Ptn*‐Tg mice, ethanol seems to modify PNNs intensity particularly in female mice, suggesting a possible hormonal intervention, as estrogens are known to regulate these nets [30]. Interestingly, although water drink *Ptn*‐overexpressing mice have fewer PNNs than *Ptn‐*Wt mice, they have a higher percentage of PV positive cells surrounded by PNNs. As PNNs facilitate GABAergic firing [31], this finding could explain the increase in hippocampal GABAergic transmission observed in *Ptn*‐Tg mice, leading to a decrease in long‐term potentiation (LTP) [32]. Surprisingly, ethanol decreases the number of PV cells in the dentate gyrus of *Ptn*‐Tg mice, despite being surrounded by PNNs. Interestingly, ethanol‐induced reduction in PNN intensity is particularly evident around PV+ neurons, possibly making these neurons more susceptible to the effects of alcohol. Still, the interpretation of data from a constitutive overexpression mouse model must be taken with caution due to its inherent limitations, including potential variability in expression levels across subjects [33] and compensatory mechanisms that may occur over time [34].

In summary, this work highlights the relevance of PTN and its biochemical pathways to modulate adolescent ethanol intake and ethanol‐induced effects on hippocampal neurogenesis, GABAergic neurons, and PNNs remodeling. Moreover, the data presented here suggest that *Ptn* overexpression prevents microglial activation induced by IAE during adolescence.

## Author Contributions

**Milagros Galán‐Llario:** investigation, data curation, formal analysis, writing – original draft, writing – review and editing. **María Rodríguez‐Zapata:** investigation, data curation, formal analysis. **Teresa Fontán‐Baselga:** investigation, data curation. **Héctor Cañeque‐Rufo:** investigation, data curation. **Alba García‐Guerra:** investigation. **Beatriz Fernández:** investigation. **Esther Gramage:** conceptualization, methodology, investigation, data curation, formal analysis. **Gonzalo Herradón:** conceptualization, methodology, funding acquisition, supervision, project administration, writing – original draft, writing – review and editing.

## Disclosure

The authors have nothing to report.

## Conflicts of Interest

The authors declare no conflicts of interest.

## Supporting information


**Table S1.** Three‐way ANOVA Results. Model, significance. Figure 1: *Ptn‐*Wt male water, *n* = 15; *Ptn‐*Wt female water, *n* = 15; *Ptn‐*Wt male EtOH, *n* = 20; *Ptn‐*Wt female EtOH, *n* = 16; *Ptn*‐Tg male water, *n* = 13; *Ptn*‐Tg female water, *n* = 10; *Ptn*‐Tg male EtOH, *n* = 16; *Ptn*‐Tg female EtOH, *n* = 13; *Ptn‐*Wt male sucrose, *n* = 8; *Ptn‐*Wt female sucrose, *n* = 3; *Ptn*‐Tg male sucrose, *n* = 8; *Ptn*‐Tg female sucrose, *n* = 7. Figure 2: *Ptn‐*Wt male water, *n* = 4; *Ptn‐*Wt female water, *n* = 3; *Ptn‐*Wt male EtOH, *n* = 7; *Ptn‐*Wt female EtOH, *n* = 5; *Ptn*‐Tg male water, *n* = 6; *Ptn*‐Tg female water, *n* = 6; *Ptn*‐Tg male EtOH, *n* = 8; *Ptn*‐Tg female EtOH, *n* = 6. Figure 3: *Ptn‐*Wt male water, *n* = 7; *Ptn‐*Wt female water, *n* = 4; *Ptn‐*Wt male EtOH, *n* = 5; *Ptn‐*Wt female EtOH, *n* = 5; *Ptn*‐Tg male water, *n* = 7; *Ptn*‐Tg female water, *n* = 4; *Ptn*‐Tg male EtOH, *n* = 8; *Ptn*‐Tg female EtOH, *n* = 6. Figure 4: *Ptn‐*Wt male water, *n* = 7; *Ptn‐*Wt female water, *n* = 4; *Ptn‐*Wt male EtOH, *n* = 5; *Ptn‐*Wt female EtOH, *n* = 5; *Ptn*‐Tg male water, *n* = 7; *Ptn*‐Tg female water, *n* = 4; *Ptn*‐Tg male EtOH, *n* = 8; *Ptn*‐Tg female EtOH, *n* = 6. Figure 5: *Ptn‐*Wt male water, *n* = 7; *Ptn‐*Wt female water, *n* = 4; *Ptn‐*Wt male EtOH, *n* = 5; *Ptn‐*Wt female EtOH, *n* = 5; *Ptn*‐Tg male water, *n* = 7; *Ptn*‐Tg female water, *n* = 4; *Ptn*‐Tg male EtOH, *n* = 8; *Ptn*‐Tg female EtOH, *n* = 6. Figure 6: *Ptn‐*Wt male water, *n* = 7; *Ptn‐*Wt female water, *n* = 4; *Ptn‐*Wt male EtOH, *n* = 5; *Ptn‐*Wt female EtOH, *n* = 5; *Ptn*‐Tg male water, *n* = 7; *Ptn*‐Tg female water, *n* = 4; *Ptn*‐Tg male EtOH, *n* = 8; *Ptn*‐Tg female EtOH, *n* = 6.

## Data Availability

Data will be made available on request.

## References

[1] A. C. May , J. Jacobus , A. N. Simmons , and S. F. Tapert , “A Prospective Investigation of Youth Alcohol Experimentation and Reward Responsivity in the ABCD Study,” Frontiers in Psychiatry 13 (2022): 886848, 10.3389/fpsyt.2022.886848. 36003980 PMC9393480

[2] B. Lees , L. R. Meredith , A. E. Kirkland , et al., “Effect of Alcohol Use on the Adolescent Brain and Behavior,” Pharmacology, Biochemistry and Behavior 192 (2020): 172906, 10.1016/j.pbb.2020.172906. 32179028 PMC7183385

[3] S. Tetteh‐Quarshie and M.‐L. Risher , “Adolescent Brain Maturation and the Neuropathological Effects of Binge Drink: A Critical Review,” Frontiers in Neuroscience 16 (2023): 1040049, 10.3389/fnins.2022.1040049. 36733924 PMC9887052

[4] K. M. Keyes , J. Jager , T. Mal‐Sarkar , M. E. Patrick , C. Rutherford , and D. Hasin , “Is There a Recent Epidemic of Women's Drink? A Critical Review of National Studies,” Alcoholism: Clinical and Experimental Research 43, no. 7 (2019): 1344–1359, 10.1111/acer.14082. 31074877 PMC6602861

[5] V. A. Macht , R. P. Vetreno , and F. T. Crews , “Cholinergic and Neuroimmune Signaling Interact to Impact Adult Hippocampal Neurogenesis and Alcohol Pathology Across Development,” Frontiers in Pharmacology 13 (2022): 849997, 10.3389/fphar.2022.849997. 35308225 PMC8926387

[6] L. J. Chandler , D. T. Vaughan , and J. T. Gass , “Adolescent Alcohol Exposure Results in Sex‐Specific Alterations in Conditioned Fear Learning and Memory in Adulthood,” Frontiers in Pharmacology 13 (2022): 837657, 10.3389/fphar.2022.837657. 35211024 PMC8861326

[7] F. T. Crews , D. L. Robinson , L. J. Chandler , et al., “Mechanisms of Persistent Neurobiological Changes Following Adolescent Alcohol Exposure: NADIA Consortium Findings,” Clinical and Experimental Research 43, no. 9 (2019): 1806–1822, 10.1111/acer.14154. PMC6758927 31335972

[8] F. T. Crews and R. P. Vetreno , “Neuroimmune Basis of Alcoholic Brain Damage,” International Review of Neurobiology 118 (2014): 315–357, 10.1016/B978-0-12-801284-0.00010-5. 25175868 PMC5765863

[9] R. Fernández‐Calle , M. Vicente‐Rodríguez , M. Pastor , et al., “Pharmacological Inhibition of Receptor Protein Tyrosine Phosphatase β/ζ (PTPRZ1) Modulates Behavioral Responses to Ethanol,” Neuropharmacology 137 (2018): 86–95, 10.1016/j.neuropharm.2018.04.027. 29753117 PMC6050104

[10] M. Galán‐Llario , M. Rodríguez‐Zapata , T. Fontán‐Baselga , et al., “Inhibition of RPTPβ/ζ Reduces Chronic Ethanol Intake in Adolescent Mice and Modulates Ethanol Effects on Hippocampal Neurogenesis and Glial Responses in a Sex‐Dependent Manner,” Neuropharmacology 227 (2023a): 109438, 10.1016/j.neuropharm.2023.109438. 36706907 PMC10327582

[11] C. A. Dannenhoffer , A. Gómez‐A , V. A. Macht , et al., “Impact of Adolescent Intermittent Ethanol Exposure on Interneurons and Their Surrounding Perineuronal Nets in Adulthood,” Alcoholism: Clinical and Experimental Research 46, no. 5 (2022): 759–769, 10.1111/acer.14810. 35307830 PMC9117471

[12] A. W. Lasek , “Effects of Ethanol on Brain Extracellular Matrix: Implications for Alcohol Use Disorder,” Alcoholism: Clinical and Experimental Research 40, no. 10 (2016): 2030–2042, 10.1111/acer.13200. 27581478 PMC5048555

[13] H. Carceller , Y. Gramuntell , P. Klimczak , and J. Nacher , “Perineuronal Nets: Subtle Structures With Large Implications,” Neuroscientist 29 (2022): 569–590, 10.1177/10738584221106346. 35872660

[14] M. Vicente‐Rodríguez , C. Pérez‐García , M. Ferrer‐Alcón , et al., “Pleiotrophin Differentially Regulates the Rewarding and Sedative Effects of Ethanol,” Journal of Neurochemistry 131, no. 5 (2014): 688–695, 10.1111/jnc.12841. 25073406

[15] G. Herradon , M. Pilar Ramos‐Alvarez , and E. Gramage , “Connecting Metainflammation and Neuroinflammation Through the PTN‐MK‐RPTPβ/ζ Axis: Relevance in Therapeutic Development,” Frontiers in Pharmacology 10 (2019): 1–13, 10.3389/fphar.2019.00377. 31031625 PMC6474308

[16] R. Fernández‐Calle , M. Vicente‐Rodríguez , E. Gramage , et al., “Pleiotrophin Regulates Microglia‐Mediated Neuroinflammation,” Journal of Neuroinflammation 14, no. 1 (2017): 1–10, 10.1186/s12974-017-0823-8. 28259175 PMC5336633

[17] E. Gramage , A. Putelli , M. J. Polanco , et al., “The Neurotrophic Factor Pleiotrophin Modulates Amphetamine‐Seeking Behaviour and Amphetamine‐Induced Neurotoxic Effects: Evidence From Pleiotrophin Knockout Mice,” Addiction Biology 15, no. 4 (2010): 403–412, 10.1111/j.1369-1600.2009.00202.x. 20192945

[18] A. Cressant , V. Dubreuil , J. Kong , et al., “Loss‐Of‐Function of PTPR γ and ζ, Observed in Sporadic Schizophrenia, Causes Brain Region‐Specific Deregulation of Monoamine Levels and Altered Behavior in Mice,” Psychopharmacology 234 (2017): 575–587, 10.1007/s00213-016-4490-8. 28025742

[19] X. Wang , “Pleiotrophin,” Activity and Mechanism 98 (2020): 51–89, 10.1016/bs.acc.2020.02.003. PMC7672882 32564788

[20] M. Galán‐Llario , M. Rodríguez‐Zapata , E. Gramage , et al., “Receptor Protein Tyrosine Phosphatase β/ζ Regulates Loss of Neurogenesis in the Mouse Hippocampus Following Adolescent Acute Ethanol Exposure,” Neurotoxicology 94 (2023b): 98–107, 10.1016/j.neuro.2022.11.008. 36402194

[21] M. Rodríguez‐Zapata , M. Galán‐Llario , H. Cañeque‐Rufo , et al., “Implication of the PTN/RPTPβ/ζ Signaling Pathway in Acute Ethanol Neuroinflammation in Both Sexes: A Comparative Study With LPS,” Biomedicine 11, no. 5 (2023): 1318, 10.3390/biomedicines11051318. PMC10215719 37238989

[22] M. Galán‐Llario , E. Gramage , A. García‐Guerra , et al., “Adolescent Intermittent Ethanol Exposure Decreases Perineuronal Nets in the Hippocampus in a Sex Dependent Manner: Modulation Through Pharmacological Inhibition of RPTPβ/ζ,” Neuropharmacology 247 (2024): 109850, 10.1016/j.neuropharm.2024.109850. 38295947

[23] N. Maeda , T. Nishiwaki , T. Shintani , H. Hamanaka , and M. Noda , “6B4 Proteoglycan/Phosphacan, an Extracellular Variant of Receptor‐Like Protein‐Tyrosine Phosphatase ζ/RPTPβ, Binds Pleiotrophin/Heparin‐Binding Growth‐Associated Molecule (HB‐GAM),” Journal of Biological Chemistry 271, no. 35 (1996): 21446–21452, 10.1074/jbc.271.35.21446. 8702927

[24] G. Herradón and C. Pérez‐García , “Targeting Midkine and Pleiotrophin Signalling Pathways in Addiction and Neurodegenerative Disorders: Recent Progress and Perspectives,” British Journal of Pharmacology 171, no. 4 (2014): 837–848, 10.1111/bph.12312. 23889475 PMC3925022

[25] H. Li , L. Xu , W. Jiang , et al., “Pleiotrophin Ameliorates Age‐Induced Adult Hippocampal Neurogenesis Decline and Cognitive Dysfunction,” Cell Reports 42, no. 9 (2023): 113022, 10.1016/j.celrep.2023.113022. 37610873

[26] E. C. Cope and E. Gould , “Adult Neurogenesis, Glia, and the Extracellular Matrix,” Cell Stem Cell 24, no. 5 (2019): 690–705, 10.1016/j.stem.2019.03.023. 31051133 PMC7961263

[27] M. Pascual , P. Baliño , C. M. G. Aragón , and C. Guerri , “Cytokines and Chemokines as Biomarkers of Ethanol‐Induced Neuroinflammation and Anxiety‐Related Behavior: Role of TLR4 and TLR2,” Neuropharmacology 89 (2015): 352–359, 10.1016/j.neuropharm.2014.10.014. 25446779

[28] A. W. Lasek , H. Chen , and W.‐Y. Chen , “Releasing Addiction Memories Trapped in Perineuronal Nets,” Trends in Genetics 34, no. 3 (2018): 197–208, 10.1016/j.tig.2017.12.004. 29289347 PMC5834377

[29] G. J. Eill , A. Sinha , M. Morawski , M. S. Viapiano , and R. T. Matthews , “The Protein Tyrosine Phosphatase RPTPξ/Phosphacan Is Critical for Perineuronal Net Structure,” Journal of Biological Chemistry 295, no. 4 (2020): 955–968, 10.1074/jbc.RA119.010830. 31822561 PMC6983847

[30] L. M. de Carvalho , H. Chen , M. Sutter , and A. W. Lasek , “Sexually Dimorphic Role for Insular Perineuronal Nets in Aversion‐Resistant Ethanol Consumption,” Frontiers in Psychiatry 14 (2023), 10.3389/fpsyt.2023.1122423. PMC10011443 36926460

[31] P. Klimczak , A. Rizzo , E. Castillo‐Gómez , et al., “Parvalbumin Interneurons and Perineuronal Nets in the Hippocampus and Retrosplenial Cortex of Adult Male Mice After Early Social Isolation Stress and Perinatal NMDA Receptor Antagonist Treatment,” Frontiers in Synaptic Neuroscience 13 (2021): 733989, 10.3389/fnsyn.2021.733989. 34630066 PMC8493248

[32] C. González‐Castillo , D. Ortuño‐Sahagún , C. Guzmán‐Brambila , M. Pallàs , and A. E. Rojas‐Mayorquín , “Pleiotrophin as a Central Nervous System Neuromodulator, Evidences From the Hippocampus,” Frontiers in Cellular Neuroscience 8 (2015): 443, 10.3389/fncel.2014.00443. 25620911 PMC4287103

[33] S. Lange and J. M. Inal , “Animal Models of Human Disease,” International Journal of Molecular Sciences 24, no. 21 (2023): 21, 10.3390/ijms242115821. PMC10650829 37958801

[34] J. C. Walrath , J. J. Hawes , T. V. Dyke , and K. M. Reilly , “Genetically Engineered Mouse Models in Cancer Research,” Advances in Cancer Research 106 (2010): 113, 10.1016/S0065-230X(10)06004-5. 20399958 PMC3533445

