# Supplementary material for: Pleiotrophin Overexpression Reduces Adolescent Ethanol Consumption and Modulates Ethanol‐Induced Glial Responses and Changes in the Perineuronal Nets in the Mouse Hippocampus
Source: CNS Neurosci Ther. 2024 Dec 9;30(12):e70159. doi: 10.1111/cns.70159 (PMC11628725; doi:10.1111/cns.70159)
Supplement: Supplementary file 1 — Table S1. Three‐way ANOVA Results. Model, significance. Figure 1: Ptn‐Wt male water, n = 15; Ptn‐Wt female water, n = 15; Ptn‐Wt male EtOH, n = 20; Ptn‐Wt female EtOH, n = 16; Ptn‐Tg male water, n = 13; Ptn‐Tg female water, n = 10; Ptn‐Tg male EtOH, n = 16; Ptn‐Tg female EtOH, n = 13; Ptn‐Wt male sucrose, n = 8; Ptn‐Wt female sucrose, n = 3; Ptn‐Tg male sucrose, n = 8; Ptn‐Tg female sucrose, n = 7. Figure 2: Ptn‐Wt male water, n = 4; Ptn‐Wt female water, n = 3; Ptn‐Wt male EtOH, n = 7; Ptn‐Wt female EtOH, n = 5; Ptn‐Tg male water, n = 6; Ptn‐Tg female water, n = 6; Ptn‐Tg male EtOH, n = 8; Ptn‐Tg female EtOH, n = 6. Figure 3: Ptn‐Wt male water, n = 7; Ptn‐Wt female water, n = 4; Ptn‐Wt male EtOH, n = 5; Ptn‐Wt female EtOH, n = 5; Ptn‐Tg male water, n = 7; Ptn‐Tg female water, n = 4; Ptn‐Tg male EtOH, n = 8; Ptn‐Tg female EtOH, n = 6. Figure 4: Ptn‐Wt male water, n = 7; Ptn‐Wt female water, n = 4; Ptn‐Wt male EtOH, n = 5; Ptn‐Wt female EtOH, n = 5; Ptn‐Tg male water, n = 7; Ptn‐Tg female water, n = 4; Ptn‐Tg male EtOH, n = 8; Ptn‐Tg female EtOH, n = 6. Figure 5: Ptn‐Wt male water, n = 7; Ptn‐Wt female water, n = 4; Ptn‐Wt male EtOH, n = 5; Ptn‐Wt female EtOH, n = 5; Ptn‐Tg male water, n = 7; Ptn‐Tg female water, n = 4; Ptn‐Tg male EtOH, n = 8; Ptn‐Tg female EtOH, n = 6. Figure 6: Ptn‐Wt male water, n = 7; Ptn‐Wt female water, n = 4; Ptn‐Wt male EtOH, n = 5; Ptn‐Wt female EtOH, n = 5; Ptn‐Tg male water, n = 7; Ptn‐Tg female water, n = 4; Ptn‐Tg male EtOH, n = 8; Ptn‐Tg female EtOH, n = 6. [file CNS-30-e70159-s001.docx]

| **Supplementary Table 1A** | | | | | | | | | | | | | | | | | | | | | |  |  |
| --- | --- | --- | --- | --- | --- | --- | --- | --- | --- | --- | --- | --- | --- | --- | --- | --- | --- | --- | --- | --- | --- | --- | --- |
| **Measure (Fig.1 Drinking behavior)** | **Day** | | | **Sex** | | | **Genotype** | | | **Day x Sex** | | | **Day x Genotype** | | | **Sex x Genotype** | | | **Day x Sex x Treatm.** | | |  |  |
|  | **Model** | | **Sig.** | **Model** | | **Sig.** | **Model** | | **Sig.** | **Model** | | **Sig.** | **Model** | | **Sig.** | **Model** | | **Sig.** | **Model** | | **Sig.** | |  |
| EtOH intake (g/kg/24h) (b) | ***F*_8, 484_ = 4.607** | | ***p* < .0001** | ***F*_1, 61_ = 4.071** | | ***p* = .0480** | ***F*_1, 49_ = 19.45** | | ***p* < .0001** | ***F*_8, 484_ = 3.664** | | ***p* = .0004** | ***F*_8, 484_ = 3.380** | | ***p* =.0009** | *F*_1, 61_ = .9226 | | *p* = .3406 | *F*_8, 484_ = .5493 | | *p* = .8192 | |  |
| EtOH intake (g/kg) (c) | ***F*_11, 671_ = 426.8** | | ***p* < .0001** | *F*_1, 61_ = 2.782 | | *p* = .1005 | ***F*_1, 61_ = 24.04** | | ***p* < .0001** | *F*_11, 671_ = 1.637 | | *p* = .0842 | ***F*_11, 671_ = 16.91** | | ***p* < .0001** | *F*_1, 61_ = .4771 | | *p* = .4923 | *F*_11, 671_ = .7543 | | *p* = .6861 | |  |
| Preference score (d) | ***F*_8, 488_ = 4.855** | | ***p* < .0001** | *F*_1, 61_ = .7891 | | *p* = .3779 | ***F*_1, 61_ =16.82** | | ***p* = .0001** | ***F*_8, 488_ = 2.103** | | ***p* = .0341** | *F*_8, 488_ = 1.703 | | *p* = .0951 | *F*_1, 61_ = .04661 | | *p* = .8298 | *F*_8, 488_ = .5442 | | *p* = .8232 | |  |
| Water intake (ml/kg/24h) (e) | | *F*_11, 531_ = 1.220 | | *p* = .2703 | ***F*_1, 49_ = 4.672** | | ***p* = .0356** | ***F*_1, 49_ = 24.43** | | ***p* < .0001** | *F*_11, 531_ = .4915 | | *p* = .9089 | *F*_11, 531_ = .1.191 | | *p* = .2897 | *F*_1, 49_ = .8823 | | *p* = .3522 | *F*_11, 531_ = .4924 | | *p* = .9083 | |
| Total Fluid consumption (ml/kg/24h) (f) | *F*_11, 663_ = .7658 | | *p* = .6745 | *F*_1, 61_ = 2.247 | | *p* = .1391 | *F*_1, 61_ =.5729 | | *p* = .4520 | *F*_11, 663_ = .9315 | | *p* = .5092 | *F*_11, 663_ = .9151 | | *p* = .5251 | *F*_1, 61_ = 2.195 | | *p* = .1436 | *F*_11, 663_ = 1.509 | | *p* = .1234 | |  |
|  |  | |  |  | |  |  | |  |  | |  |  | |  |  | |  |  | |  | |  |
| **Supplementary Table 1B** | | | | | | | | | | | | | | | | | | | | | | |  |
| **Measure (Fig.2 Ptn levels)** | **Sex** | | | **Genotype** | | | **Drinking** | | | **Sex x Genotype** | | | **Sex x Drink** | | | **Genotype x Drink** | | | **Sex x Genotype x Drink** | | | |  |
|  | **Model** | | **Sig.** | **Model** | | **Sig.** | **Model** | | **Sig.** | **Model** | | **Sig.** | **Model** | | **Sig.** | **Model** | | **Sig.** | **Model** | | **Sig.** | |  |
| Ptn levels (a) | *F*_1, 34_ = .3637 | | *p* = .5504 | ***F*_1, 34_ = 9.3430** | | ***p* = .0042** | ***F*_1, 34_ = 7.604** | | ***p* = .0093** | *F*_1, 34_ = .0023 | | *p* = .9620 | *F*_1, 35_ = .0360 | | *p* = .8507 | ***F*_1, 35_ = 5.660** | | ***p* = .0231** | *F*_1, 35_ = .2038 | | *p* = .6546 | |  |
|  | | | | | | | | | | | | | | | | | | | | | | |  |
| **Supplementary Table 1C** | | | | | | | | | | | | | | | | | | | | | |  |  |
| **Measure (Fig.3 Neurogenesis)** | **Sex** | | | **Genotype** | | | **Drinking** | | | **Sex x Genotype** | | | **Sex x Drinking** | | | **Genotype x Drinking** | | | **Sex x Genotype x Drinking** | | |  |  |
|  | **Model** | | **Sig.** | **Model** | | **Sig.** | **Model** | | **Sig.** | **Model** | | **Sig.** | **Model** | | **Sig.** | **Model** | | **Sig.** | **Model** | | **Sig.** | |  |
| %DCX+/GCL (b) | *F*_1, 118_ = .3399 | | *p* = .5610 | ***F*_1, 118_ = 303.8** | | ***p* < .0001** | ***F*_1, 118_ = 17.30** | | ***p* < .0001** | *F*_1, 118_ = 1.050 | | *p* = .3076 | *F*_1,118_ = 3.042 | | *p* =.0837 | *F*_1, 118_ = 1.315 | | *p* = .2538 | *F*_1, 118_ = 2.177 | | *p* = .1427 | |  |
|  |  | |  |  | |  |  | |  |  | |  |  | |  |  | |  |  | |  | |  |
| **Supplementary Table 1D** | | | | | | | | | | | | | | | | | | | | | | |  |
| **Measure (Fig. 4 Microglia)** | **Sex** | | | **Genotype** | | | **Drinking** | | | **Sex x Genotype** | | | **Sex x Drink** | | | **Genotype x Drink** | | | **Sex x Genotype x Drink** | | | |  |
|  | **Model** | | **Sig.** | **Model** | | **Sig.** | **Model** | | **Sig.** | **Model** | | **Sig.** | **Model** | | **Sig.** | **Model** | | **Sig.** | **Model** | | **Sig.** | |  |
| Iba1+ cells count (b) | *F*_1, 120_ = .2410 | | *p* = .6243 | ***F*_1, 120_ = 33.26** | | ***p* < .0001** | ***F*_1, 120_ = 40.55** | | ***p* < .0001** | ***F*_1, 120_ = 16.74** | | ***p* < .0001** | ***F*_1, 120_ = 7.651** | | ***p* < .0001** | ***F*_1, 120_ = 5.965** | | ***p* = .0160** | ***F*_1, 120_ = 12.92** | | ***p* = .0005** | |  |
| Iba1+ average size (c) | *F*_1, 205_ = .2212 | | *p* = .6386 | ***F*_1, 205_ = 75.33** | | ***p* < .0001** | ***F*_1, 205_ = 7.743** | | ***p* = .0059** | *F*_1, 205_ = .7391 | | *p* = .3910 | *F*_1, 205_ = .4864 | | *p* = .4863 | ***F*_1, 205_ = 51.53** | | ***p* < .0001** | *F*_1, 205_ = .5769 | | *p* = .4484 | |  |
| Iba1+ circularity (d) | *F*_1, 205_ = 2.334 | | *p* = .1281 | ***F*_1, 205_ = 430.8** | | ***p* < .0001** | *F*_1, 205_ = .1609 | | *p* = .6887 | *F*_1, 205_ = .03357 | | *p* = .8548 | *F*_1, 205_ = .3928 | | *p* = .5315 | *F*_1, 205_ = .3731 | | *p* = .5420 | *F*_1, 205_ = 2.024 | | *p* = .1563 | |  |
| Iba1+ body size (e ) | *F*_1, 274_ = .6827 | | *p* = .4094 | ***F*_1, 274_ = 25.53** | | ***p* < .0001** | *F*_1, 274_ = 1.365 | | *p* = .2437 | *F*_1, 274_ = .2678 | | *p* = .6052 | *F*_1, 274_ = 1.018 | | *p* = .3138 | *F*_1, 274_ = .05269 | | *p* = .8186 | *F*_1, 274_ = .5533 | | *p* = .4576 | |  |
| Iba1+ ramification length (f) | *F*_1, 274_ = 3.769 | | *p* = .0532 | *F*_1, 274_ = .097333 | | *p* = .7553 | ***F*_1, 274_ = 15.77** | | ***p* < .0001** | *F*_1, 274_ = 3.176 | | *p* = .0759 | *F*_1, 274_ = 1.040 | | *p* = .3086 | ***F*_1, 274_ = 19.37** | | ***p* < .0001** | *F*_1, 274_ = .8891 | | *p* = .3466 | |  |
| Iba1+ complexity(g) | *F*_1, 274_ = 2.310 | | *p* = .130 | *F*_1, 274_ = .076 | | *p* = .783 | ***F*_1, 274_ = 15.708** | | ***p* < .0001** | ***F*_1, 274_ = 5.956** | | ***p* = .015** | *F*_1, 274_ = 1.981 | | *p* = .160 | ***F*_1, 274_ = 19.292** | | ***p* < .0001** | *F*_1, 274_ = .294 | | *p* = .588 | |  |
|  |  | |  |  | |  |  | |  |  | |  |  | |  |  | |  |  | |  | |  |
| **Supplementary Table 1E** | | | | | | | | | | | | | | | | | | | | | | |  |
| **Measure (Fig.5 Astrocytes)** | **Sex** | | | **Genotype** | | | **Drinking** | | | **Sex x Genotype** | | | **Sex x Drink** | | | **Genotype x Drink** | | | **Sex x Genotype x Drink** | | | |  |
|  | **Model** | **Sig.** | | **Model** | **Sig.** | | **Model** | **Sig.** | | **Model** | **Sig.** | | **Model** | **Sig.** | | **Model** | **Sig.** | | **Model** | **Sig.** | | |  |
| GFAP+ cells count (a) | *F*_1, 118_ = 1.199 | *p* = .2757 | | ***F*_1, 118_ = 127.8** | ***p* < .0001** | | *F*_1, 118_ = 2.208 | *p* = .1400 | | *F*_1, 118_ = .0005022 | *p* = .9822 | | ***F*_1, 118_ = 6.322** | ***p* = .0133** | | *F*_1, 118_ = .4414 | *p* = .5078 | | ***F*_1, 118_ = 5.578** | ***p* = .0198** | | |  |
|  | | | | | | | | | | | | | | | | | | | | | | |  |
| **Supplementary Table 1F** | | | | | | | | | | | | | | | | | | | | | | |  |
| **Measure (Fig.6 PNNs)** | **Sex** | | | **Genotype** | | | **Drinking** | | | **Sex x Genotype** | | | **Sex x Drink** | | | **Genotype x Drink** | | | **Sex x Genotype x Drink** | | | |  |
|  | **Model** | **Sig.** | | **Model** | **Sig.** | | **Model** | **Sig.** | | **Model** | **Sig.** | | **Model** | **Sig.** | | **Model** | **Sig.** | | **Model** | **Sig.** | | |  |
| WFA Intensity (b) | *F*_1, 84_ = .3053 | *p* = .5820 | | ***F*_1, 84_= 20.59** | ***p* < .0001** | | ***F*_1, 84_ = 11.27** | ***p* = .0012** | | *F*_1, 84_ = .07098 | *p* = .7906 | | *F*_1, 84_ = .4810 | *p* = .4899 | | *F*_1, 84_ = .007401 | *p* = .9316 | | ***F*_1, 84_ = 5.101** | ***p* = .0265** | | |  |
| WFA number of cells (c ) | *F*_1, 76_ = .4098 | *p* = .5240 | | ***F*_1, 76_ = 78.19** | ***p* < .0001** | | *F*_1, 76_ = .3881 | *p* = .5352 | | *F*_1, 76_ = 1.828 | *p* = .1804 | | *F*_1, 76_ = .1458 | *p* = .7036 | | *F*_1, 76_ = 2.428 | *p* = .1234 | | *F*_1, 76_ = .3286 | *p* = .5682 | | |  |
| PV Number of cells (d) | *F*_1, 76_ = .1270 | *p* = .7225 | | ***F*_1, 76_ = 4.621** | ***p* = .0347** | | *F*_1, 76_ = .2159 | *p* = .6434 | | ***F*_1, 76_ = 8.183** | ***p* = .0054** | | ***F*_1, 76_ = 4.178** | ***p* = .00443** | | ***F*_1, 76_ = 20.71** | ***p* < .0001** | | ***F*_1, 76_ = 10.70** | ***p* = .0016** | | |  |
| **WFA+** PV+ Cells (e ) | *F*_1, 77_ = .3917 | *p* = .5333 | | ***F*_1, 77_ = 10.94** | ***p* = .0014** | | *F*_1, 77_ = 1978 | *p* = .6577 | | ***F*_1, 77_ =5.197** | ***p* = .0254** | | *F*_1, 77_ = 1.216 | *p* = .2736 | | ***F*_1, 77_ = 14.62** | ***p* = .0003** | | *F*_1, 77_ = 3.276 | *p* = .0742 | | |  |
| **WFA+** PV- Cells (e ) | *F*_1, 76_ = .5158 | *p* = .4749 | | ***F*_1, 76_ = 98.37** | ***p* < .0001** | | *F*_1, 76_ = .09628 | *p* = .7572 | | *F*_1, 76_ = .0004716 | *p* = .9827 | | *F*_1, 76_ = .1567 | *p* = .6934 | | *F*_1, 76_ = .5645 | *p* = .4548 | | *F*_1, 76_ = .1964 | *p* = .6589 | | |  |
| **PV+** WFA- Cells (e ) | *F*_1, 77_ = .05426 | *p* = .8164 | | *F*_1, 77_ = .06413 | *p* = .8008 | | *F*_1, 77_ = 1.064 | *p* = .3056 | | ***F*_1, 77_ = 6.178** | ***p* = .0151** | | ***F*_1, 77_ = 4.233** | ***p* = .0430** | | ***F*_1, 77_ = 15.79** | ***p* = .0002** | | ***F*_1, 77_ = 13.36** | ***p* = .0005** | | |  |
| **WFA+** PV+ Intensity (f) | *F*_1, 65_ = .2269 | *p* = .6354 | | ***F*_1, 65_ = 5.380** | ***p* = .0235** | | ***F*_1, 65_ = 8.724** | ***p* = .0044** | | *F*_1, 65_ = 1.712 | *p* = .1954 | | *F*_1, 65_ = .1692 | *p* = .6822 | | *F*_1, 65_ = 1.608 | *p* = .2093 | | *F*_1, 65_ = .6314 | *p* = .4297 | | |  |
| **WFA+** PV- Intensity(g) | *F*_1, 66_ = 1.936 | *p* = .1688 | | ***F*_1, 66_ = 17.59** | ***p* < .0001** | | ***F*_1, 66_ = 6.397** | ***p* = .0138** | | *F*_1, 66_ = .005556 | *p* = .9408 | | *F*_1, 66_ = 1.544 | *p* = .2184 | | *F*_1, 66_ = 6292 | *p* = .4305 | | *F*_1, 66_ = 2.625 | *p* = .1099 | | |  |

**Supplementary Table 1: Three-way ANOVA Results.** Model, significance. Figure 1: *Ptn-*Wt male water, n= 15; *Ptn-*Wt female water, n= 15; *Ptn-*Wt male EtOH, n= 20; *Ptn-*Wt female EtOH, n= 16; *Ptn*-Tg male water, n=13; *Ptn*-Tg female water, n=10; *Ptn*-Tg male EtOH, n=16; *Ptn*-Tg female EtOH, n=13; *Ptn-*Wt male sucrose, n= 8; *Ptn-*Wt female sucrose, n= 3; *Ptn*-Tg male sucrose, n= 8; *Ptn*-Tg female sucrose, n= 7. Figure 2: *Ptn-*Wt male water, n= 4; *Ptn-*Wt female water, n= 3; *Ptn-*Wt male EtOH, n= 7; *Ptn-*Wt female EtOH, n= 5; *Ptn*-Tg male water, n=6; *Ptn*-Tg female water, n=6; *Ptn*-Tg male EtOH, n=8; *Ptn*-Tg female EtOH, n=6. Figure 3: *Ptn-*Wt male water, n= 7; *Ptn-*Wt female water, n= 4; *Ptn-*Wt male EtOH, n= 5; *Ptn-*Wt female EtOH, n= 5; *Ptn*-Tg male water, n=7; *Ptn*-Tg female water, n=4; *Ptn*-Tg male EtOH, n=8; *Ptn*-Tg female EtOH, n=6. Figure 4: *Ptn-*Wt male water, n= 7; *Ptn-*Wt female water, n= 4; *Ptn-*Wt male EtOH, n= 5; *Ptn-*Wt female EtOH, n= 5; *Ptn*-Tg male water, n=7; *Ptn*-Tg female water, n=4; *Ptn*-Tg male EtOH, n=8; *Ptn*-Tg female EtOH, n=6. Figure 5: *Ptn-*Wt male water, n= 7; *Ptn-*Wt female water, n= 4; *Ptn-*Wt male EtOH, n= 5; *Ptn-*Wt female EtOH, n= 5; *Ptn*-Tg male water, n=7; *Ptn*-Tg female water, n=4; *Ptn*-Tg male EtOH, n=8; *Ptn*-Tg female EtOH, n=6. Figure 6: *Ptn-*Wt male water, n= 7; *Ptn-*Wt female water, n= 4; *Ptn-*Wt male EtOH, n= 5; *Ptn-*Wt female EtOH, n= 5; *Ptn*-Tg male water, n=7; *Ptn*-Tg female water, n=4; *Ptn*-Tg male EtOH, n=8; *Ptn*-Tg female EtOH, n=6.
